# Effects of temperature on bacterial microbiome composition in *Ixodes scapularis* ticks

**DOI:** 10.1002/mbo3.719

**Published:** 2018-09-21

**Authors:** Santosh Thapa, Yan Zhang, Michael S. Allen

**Affiliations:** ^1^ Tick Borne Disease Research Laboratory Department of Microbiology, Immunology and Genetics University of North Texas Health Science Center Fort Worth Texas

**Keywords:** 16S rRNA, blacklegged tick, ecology, *Ixodes scapularis*, microbiome, tick‐temperature behavior

## Abstract

*Ixodes scapularis*, the blacklegged deer tick, is the principal vector of Lyme disease in North America. Environmental factors are known to influence regional and seasonal incidence of Lyme disease and possibly the endemicity of the disease to the northeastern and upper mid‐western regions of the United States. With a goal to understand the impact of environmental temperature on microbial communities within the tick, we investigated the bacterial microbiome of colony‐reared *I. scapularis* ticks statically incubated at different temperatures (4, 20, 30, and 37°C) at a constant humidity in a controlled laboratory setting by comparison of sequenced amplicons of the bacterial 16S V4 rRNA gene to that of the untreated baseline controls. The microbiomes of colony‐reared *I. scapularis* males were distinct than that of females, which were entirely dominated by *Rickettsia*. In silico removal of *Rickettsia* sequences from female data revealed the underlying bacterial community, which is consistent in complexity with those seen among male ticks. The bacterial community composition of these ticks changes upon incubation at 30°C for a week and 37°C for more than 5 days. Moreover, the male ticks incubated at 30 and 37°C exhibited significantly different bacterial diversity compared to the initial baseline microbiome, and the change in bacterial diversity was dependent upon duration of exposure. *Rickettsia*‐free data revealed a significantly different bacterial diversity in female ticks incubated at 37°C compared to that of 4 and 20°C treatments. These results provide experimental evidence that environmental temperature can impact the tick bacterial microbiome in a laboratory setting.

## 1 INTRODUCTION

*Ixodes scapularis* (commonly known as the “blacklegged” or “deer” tick) is the primary North American vector of the spirochete bacterium *Borrelia burgdorferi*, the etiological agent of Lyme disease and the most commonly reported vectorborne illness in the United States (Burgdorfer et al., 1982; CDC, 2015a, 2017a,b; Schwartz, Hinckley, Mead, Hook, & Kugeler, 2017). The tick is also a vector for a number of other human pathogens, including *Anaplasma phagocytophilum*,* Babesia microti*, and Powassan virus (CDC, 2017a; Ebel, 2010; Piesman & Eisen, 2008). In addition, these ticks carry a variety of commensal and endosymbiotic bacteria of unknown pathogenicity (Bonnet, Binetruy, Hernández‐Jarguín, & Duron, 2017; Narasimhan & Fikrig, 2015).

Recent studies have increasingly shown important roles of the tick microbiome in vector competence and pathogen transmission dynamics for many tickborne diseases (Bonnet et al., 2017; Clay & Fuqua, 2010; Narasimhan & Fikrig, 2015; Zolnik, Prill, Falco, Daniels, & Kolokotronis, 2016). Studies have found that endosymbiotic bacteria within ticks play crucial roles not only in reproductive fitness (Zolnik et al., 2016) and provision of nutrients (Smith, Driscoll, Gillespie, & Raghavan, 2015), but can also influence pathogen acquisition, virulence, and transmission (Gall et al., 2016). One prior study showed that the bacterial microbiomes of *Ixodes* ticks varied by geographical origin, species, sex, and life stages (Van Treuren et al., 2015), yet the factors that drive the composition and diversity of the tick microbial community have not been thoroughly investigated. Previous work from our laboratory has demonstrated that the microbial survivorship and diversity of bacteria within *Amblyomma americanum* ticks (the lone star tick, a three‐host‐tick belonging to the same hard tick family as that of *I. scapularis*) are partially dependent on environmental variables (Menchaca et al., 2013). However, no published studies have systematically assessed the direct effects of environmental factors such as temperature on the composition and diversity of the microbiomes of *I. scapularis* ticks.

Ticks are ectothermic (Ogden et al., 2004; Sonenshine & Roe, 2014) and spend the majority of their life cycle (98% of the 2‐year life cycle in *I. scapularis)* off host (Fish, 1993). Different species of bacteria inhabiting ticks may also be expected to exhibit differences in optimal growth temperatures (Sonenshine & Roe, 2014). To better understand the effects of environmental temperature on the microbial communities of ticks, we investigated the bacterial microbiomes of colony‐reared *I. scapularis* ticks statically incubated at different temperatures (4, 20, 30, and 37°C) at a constant relative humidity (RH) of >80% in a controlled laboratory setting, by comparison of sequenced amplicons of the hypervariable region four (V4) of the bacterial 16S rRNA gene to that of the untreated baseline controls. To provide a more uniform microbiome population for the experiment, we used *I. scapularis* adult ticks reared in a single facility. The resultant data provide information regarding the plasticity of the tick microbiome and the impact of environmental temperature on the underlying bacterial community compositions, which may provide insight into disease risk and aid in establishing effective interventions to combat tickborne diseases.

## 2 MATERIALS AND METHODS

### 2.1 Tick samples, processing, and incubation

Approximately 4‐month‐old live unfed *I. scapularis* adults (*n* = 90) were purchased from a colony maintained at the Tick Rearing Facility at Oklahoma State University (OSU), Stillwater, OK, USA. These adult ticks had not been fed a blood meal, but nymphs were previously fed on pathogen‐free New Zealand white rabbits (*Oryctolagus cuniculus*) in accordance with the OSU Institutional Animal Care and Use Committee (IACUC) Animal Use Protocol No. AG‐12‐14 (Lisa Coburn, personal communication). The adult ticks in the facility were held at ~22.8°C at a constant relative humidity of ~90% with a photoperiod of 15 light/9 dark (Lisa Coburn, personal communication).

The live ticks were sent overnight to our laboratory in plastic containers with moist paper towels to maintain a humid environment. Immediately after receiving the ticks, individuals were sorted by sex (45 males, 45 females), based on the Centers for Disease Control and Prevention (CDC) morphological classification criteria (CDC, 2015b). Ten individual ticks (five males and five females) were immediately stored at −20°C in separate sandwich bags (16.5 cm × 8.2 cm) until DNA extraction, and these ticks served as a baseline control for the study. Eighty remaining ticks were placed into eight separate autoclaved glass mason jars (*n* = 10 per jar, either all males or all females) containing strips of sterile meshed fabric to provide housing material for the ticks and to reduce condensation. The tops of the jars were also covered with fabric to allow the conditions in the jar to equilibrate with that of the environmental chambers in which they were placed while containing the ticks.

Appropriate saturated salt solutions were used to maintain a constant relative humidity of 80‐95% at each given temperature as follows: ammonium sulfate [(NH4)_2_SO_4_], 4°C; potassium chloride (KCl), 20 and 30°C; and potassium sulfate (K_2_SO_4_), 37°C (MiTeGen, 2016; Rockland, 1960; Winston & Bates, 1960).

**Figure 1:**
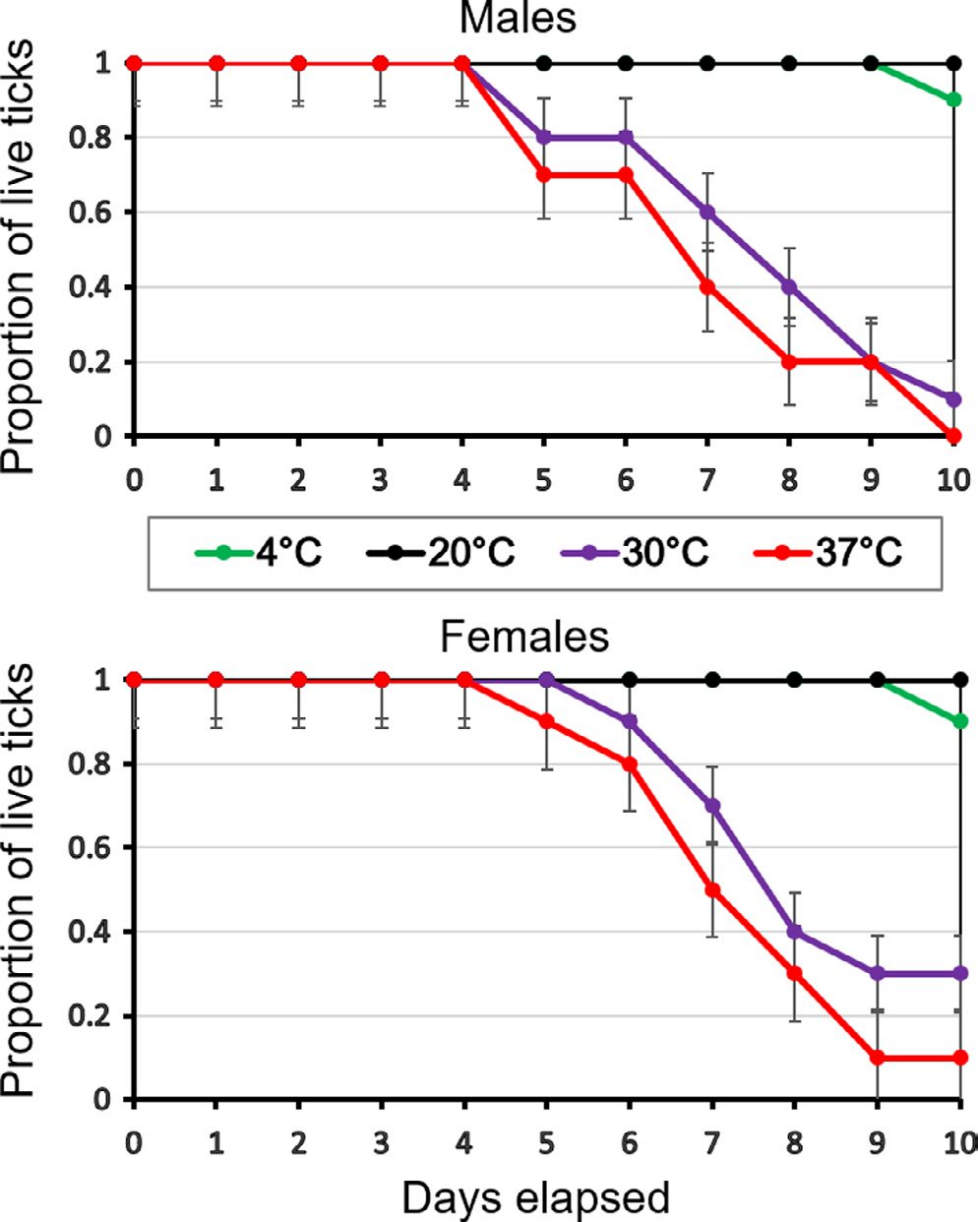
Survival of *Ixodes scapularis* males (upper panel) and females (lower panel) under different incubation temperatures

The mason jars containing ticks, along with vessels of appropriate saturated salt solutions, were then placed in four different treatment conditions: two static incubators set at 37 and 30°C, respectively, a drawer from the laboratory refrigerator (4°C), and a plastic container placed at room temperature in the laboratory (20°C). To maintain a constant relative humidity, jars holding ticks and vials of salt solutions were first placed into three separate Styrofoam boxes before putting them at 4, 30, and 37°C. The plastic container set at room temperature was further sealed with Saran wrap to maintain constant humidity. Temperature and humidity in different environmental chambers were monitored throughout the experiment using four Onset HOBO Temp/RH Data Loggers (Onset Computer Corp., Cape Cod, Massachusetts, USA) with a logging interval of every 30 min. Dead ticks, if present, were removed from the chamber every day, and placed at −20°C in separate sealed plastic containers until DNA extraction. By day 10, only 15% of the total ticks were alive at higher temperatures (30 and 37°C combined) (see Figure 1), and thus, we terminated the experiment by preserving all the remaining ticks at −20°C until processing.

### 2.2 Selection of incubation temperature and humidity

Temperature selection was determined by comparison of geographical distribution maps of *I. scapularis* (CDC, 2017a) and reported cases of Lyme disease in the United States (CDC, 2017b; Schwartz et al., 2017), the summer and winter temperatures of the areas that possessed the same vectors but with differing rates of Lyme infection (NOAA, 2017), published studies (Estrada‐Pena, Ayllon, & de la Fuente, 2012; Hubalek, Halouzka, & Heroldova, 1998), and feasibility within the laboratory. The 4°C corresponds to the average minimum temperature across the US South Climatic Region during October to March (peak activity period of adult *I. scapularis* in Texas) (Santelises, 2013) for the years 2010–2017 (Supporting Information Figure S1a), while 37°C is the maximum average temperature of the US South Climatic Region during August of the same years (Supporting Information Figure S1b) (NOAA, 2017). A temperature of 20°C corresponds to an average daily temperature across the Upper Midwest (Supporting Information Figure S1c) and northeast (Supporting Information Figure S1d) states of the United States during June–July of the years 2010–2017 (NOAA, 2017), which in turn corresponds to the peak nymphal activity in the region (CDC, 2017b). The humidity of >80% was chosen because high relative humidity is critical for survival and activity of *I. scapularis* ticks, with optimal level ranging from 80% to 95% depending on temperature (Rodgers, Zolnik, & Mather, 2007; Stafford, 1994; Troughton & Levin, 2007).

### 2.3 DNA extraction from ticks

Genomic DNA was extracted individually from 90 adult *I. scapualris* ticks using the Mag‐Bind^®^ Plant DNA Plus Kit (Omega Bio‐tek, Norcross, GA) following the manufacturer's protocol with minor modifications as described below. Prior to extraction, ticks were surface sterilized by dipping in 10% (v/v) sodium hypochlorite solution for 30 s, followed by rinsing with molecular biology grade water for 1 min (Greay et al., 2018; Menchaca et al., 2013). Then, each tick was thoroughly air‐dried on a microscopic slide, cut into at least eight different sections with a sterile scalpel, and the entire tick was placed in a 2‐ml screw‐capped FastPrep tube (MP Biomedicals, LLC., Santa Ana, CA) containing 550 μl CSPL^®^ buffer (Omega Bio‐Tek, Norcross, GA) and about 8–10 sterile 2.8‐mm ceramic beads (Mo Bio Laboratories Inc., Carlsbad, CA). The entire tick was pulverized at a speed of 7 m/s for 60 s (three cycles) using the FastPrep‐24^™^ 5G Instrument (MP Biomedicals, LLC., Santa Ana, CA) and subsequently incubated at 56°C for 2 hr. Resultant genomic DNA was quantified using a high‐sensitivity dsDNA assay on Qubit^®^ 2.0 fluorometer (Invitrogen, Carlsbad, CA) and stored at −20°C until future use. A “blank extraction,” containing only the extraction reagents and beads, was also prepared for every set of DNA extractions. In total, eight extraction reagent controls were performed during the experiments. One representative “blank extraction control” was processed for sequencing along with the samples to access the background contamination.

### 2.4 Tick mitochondrial 16S rRNA gene amplification

As a sample positive control, each DNA extract was PCR amplified with 16S−1 and 16S+2 primers specific for tick mitochondrial 16S rRNA gene (Black & Piesman, 1994), using 5 μl of template DNA in a 25 μl reaction mixture containing 5 μl 10× ThermoPol^®^ reaction buffer (New England Biolabs, Inc., Ipswich, MA), 2.5 μl 10× bovine serum albumin, 2 μl of 2.5 mM DNTPs, 0.5 μl forward primer, 0.5 μl reverse primer, 0.25 μl *Taq* DNA polymerase, and 11.75 μl molecular biology grade water. Amplification was performed in a Bio‐Rad C1000 Touch^™^ thermal cycler (Bio‐Rad Laboratories, Inc., Hercules, CA) as follows: 10 min of initial denaturation at 94°C followed by nine cycles consisting of denaturation at 92°C for 1 min, annealing at 50°C for 1 min, and extension at 72°C for 1.30 min, then followed by 31 cycles consisting of denaturation at 92°C for 1 min, annealing at 56°C for 1 min, and extension at 72°C for 1.30 min, with a final extension at 72°C for 10 min and indefinite hold at 4°C. The PCR products were electrophoresed on a 1.5% agarose gel stained with ethidium bromide and subsequently visualized under UV light.

### 2.5 16S rRNA gene library preparation and sequencing

All samples were PCR amplified in duplicates using 515F (GTGCCAGCMGCCGCGGTAA) and 806R (GGACTACHVGGGTWTCTAAT) primers with Illumina sequencing adaptor, that target the hypervariable region four (V4) of the bacterial 16S rRNA gene, as described in the Earth Microbiome Project (EMP) 16S Illumina Amplification Protocol (Earth Microbiome Project, 2016) with minor modifications as mentioned below. In short, a master mix solution was prepared per 25 μl PCR volume with 2.5 μl 10× Accuprime^™^ PCR Buffer II (Invitrogen, Carlsbad, CA), 2.5 μl 10× Bovine Serum Albumin (New England Biolabs, Inc., Ipswich, MA), 1 μl 50 mM MgSO_4_, 0.5 μl 10 μM forward primer, 0.5 μl 10 μM reverse primer, 0.1 μl of 5 U/μl Accuprime^™^ *Taq* DNA Polymerase High Fidelity, 10 μl of template DNA, and 7.9 μl molecular biology grade water. For every master mix created, 10 μl of molecular biology grade water was used as a no‐template PCR control (NTC) and 10 μl of known *Escherichia coli* genomic DNA was used as a positive control. Amplification was carried out in a Bio‐Rad C1000 Touch^™^ thermal cycler with the following cycling parameters: an initial denaturation at 94°C for 2 min followed by 30 cycles (35 cycles for all male samples) consisting of denaturation at 94°C for 30 s, annealing at 55°C for 40 s, and extension at 68°C for 40 s, with a final extension at 68°C for 5 min and a 4°C indefinite hold. Amplicon quality was visualized under UV light after separation via gel electrophoresis (1.5% agarose). None of the NTCs produced bands in gel electrophoresis and were not processed for sequencing.

PCR products were purified using AMPure XP magnetic beads, and separate 16S libraries were prepared for each amplicon sample following the Illumina 16S metagenomic sequencing library preparation guide. Each sample was labeled with specific indices through index PCR using AccuPrime High‐fidelity DNA polymerase. The master mix solution for index PCR (per 50 μl reaction) included 5 μl 10× Accuprime^™^ PCR Buffer II, 5 μl Nextera XT Index Primer 1, 5 μl Nextera XT Index Primer 2, 0.2 μl Accuprime^™^ *Taq* DNA Polymerase High Fidelity (5U/μl), 5 μl 16S V4 PCR amplicon product, and 29.8 μl molecular grade water. The PCR parameters were as follows: an initial denaturation step at 94°C for 3 min followed by eight cycles consisting of denaturation at 94°C for 30 s, annealing at 55°C for 30 s, and extension at 68°C for 30 s, with a final extension at 68°C for 5 min and an indefinite hold at 4°C. Purified 16S libraries were quantified using a high‐sensitivity dsDNA assay on Qubit^®^ 2.0 fluorometer (Invitrogen, Carlsbad, CA) and stored at −20°C until further processing. The libraries were pooled in equimolar amounts. The final concentration of the library loaded in the MiSeq Reagent Kit v2 (Illumina Inc, San Diego, CA) was 10 pM. The MiSeq run also included a 5% PhiX DNA as an internal control for potentially low‐diversity libraries. Paired‐end (2 × 250) high‐throughput sequencing (500 cycles) was performed according to the manufacturer's recommendations on Illumina MiSeq^®^ instrument.

### 2.6 Sequence processing and analysis

Raw sequence data were processed with the mothur v1.36.1 (Schloss et al., 2009), as previously described (Kozich, Westcott, Baxter, Highlander, & Schloss, 2013). Quality‐filtered merged reads were aligned to the SILVA database (Pruesse et al., 2007) and screened for chimeras using UCHIME algorithm (Edgar, Haas, Clemente, Quince, & Knight, 2011). Sequences with 97% similarity were then grouped into operational taxonomic units (OTUs) (Schloss & Westcott, 2011) using OptiClust clustering algorithm (Westcott & Schloss, 2017) and assigned to taxonomic groups by comparison to the Greengenes reference database (version 13.8.99) (DeSantis et al., 2006; McDonald et al., 2012) using RDP classifier (Wang, Garrity, Tiedje, & Cole, 2007) in mothur 1.36.1, with default parameters. After classification, in silico deletion of *Rickettsia* sequences from the dataset was performed using the remove.lineage command in mothur. The corresponding OTUs were also removed while calculating diversity. Relative abundances of bacterial taxa in each sample were calculated and compared among groups. Taxa with a relative abundance of ≥1% in at least one sample were analyzed individually, while those with <1% relative abundance in all samples were grouped together into “Others” category. “Unclassified (phylum level)” were the OTUs that did not match to any of the sequences in the Greengenes database. Bacterial diversity was estimated from the normalized data: Each sample was rarefied to reduce the bias of uneven sequencing depth (Gihring, Green, & Schadt, 2012). Samples with all sequences were rarefied to 2,399 sequence reads, the lowest library size. The rarefaction cutoff for *Rickettsia‐*deleted datasets was 1,034 reads, to include the samples with above 1,000 reads while minimizing the number of samples being excluded. Various metrics, including observed OTUs and Shannon index, were used to estimate alpha‐diversity of all the samples, while beta‐diversity between samples was assessed using principal coordinates analysis (PCoA) plots of the weighted and unweighted UniFrac distances (Supporting Information Figure S2).

### 2.7 Characterization of *Rickettsia*

To better characterize the samples identified to contain *Rickettsia* sequences by microbiome analysis, we performed PCR using Rr.190 70P and Rr.190 602N primers for the *Rickettsia*‐specific *ompA* gene on a subset of *Rickettsia*‐positive samples. The resultant amplicons were Sanger sequencing as previously described (Mitchell et al., 2016). Obtained *ompA* sequences were compared with the NCBI GenBank nucleotide reference database using BLAST.

### 2.8 Statistical analysis

The Microsoft Excel 2016 for Windows (Microsoft Corporation, Redmond, WA, USA), XLSTAT‐Ecology (Addinsoft SARL, New York, USA), and R software (version 3.4.1) were used for statistical analyses. As our data did not meet normality assumptions (using Shapiro–Wilk test), nonparametric analyses were performed for this study. Kruskal–Wallis group significance testing was employed to analyze the bacterial community composition between various groups. In addition, comparisons of bacterial diversity (Shannon index) among more than two groups utilized a Kruskal–Wallis test, while analysis of molecular variance (AMOVA) (Kozich et al., 2013; Mothur, 2018) test was used for analyzing the UniFrac distance matrices of the female ticks after in silico removal of *Rickettsia* sequences. Shannon diversity between two groups was compared using a Mann–Whitney test. Where multiple comparison testing was performed, a Benjamini–Hochberg correction (Benjamini, 2010) was applied at a false discovery rate (FDR) of 0.05 (Weiss et al., 2017). Comparisons between bacterial community compositions were performed using a permutational multivariate analysis of variance (PERMANOVA) in the vegan package of R (Anderson & Walsh, 2013; Oksanen et al., 2017).

## 3 RESULTS

### 3.1 16S V4 sequencing results

A total of 9,431,153 sequences (≥Q30 = 81.20%) were obtained from 89 *I. scapularis* tick samples (all samples except for the 4°C male group where one sample failed to amplify) sequenced for bacterial 16S V4 rRNA gene amplicons using an Illumina MiSeq. On average, we obtained 105,968 mothur‐processed quality sequences (±41,579 standard deviation) per sample. Moreover, a total of 2,133 unique OTUs were detected in 89 samples, with an average of 90 OTUs (±37 *SD*) per sample. The blank extraction control had nine unique OTUs. The blank extraction control resulted in about 0.0005% of all quality‐filtered sequences and was excluded from further analysis. Rarefaction curves at a depth from 1,000 to 30,000 sequences suggested sufficient sequencing depth as shown by the representative curves of observed OTUs reaching a plateau (Supporting Information Figure S3) (except 4°C male tick1, and 37°C male tick1, tick2, tick3; but all of these four samples had Good's coverage of greater than 98% as shown in Supporting Information Table S1).

### 3.2 Baseline bacterial microbiome of *I. scapularis* ticks

**Figure 2:**
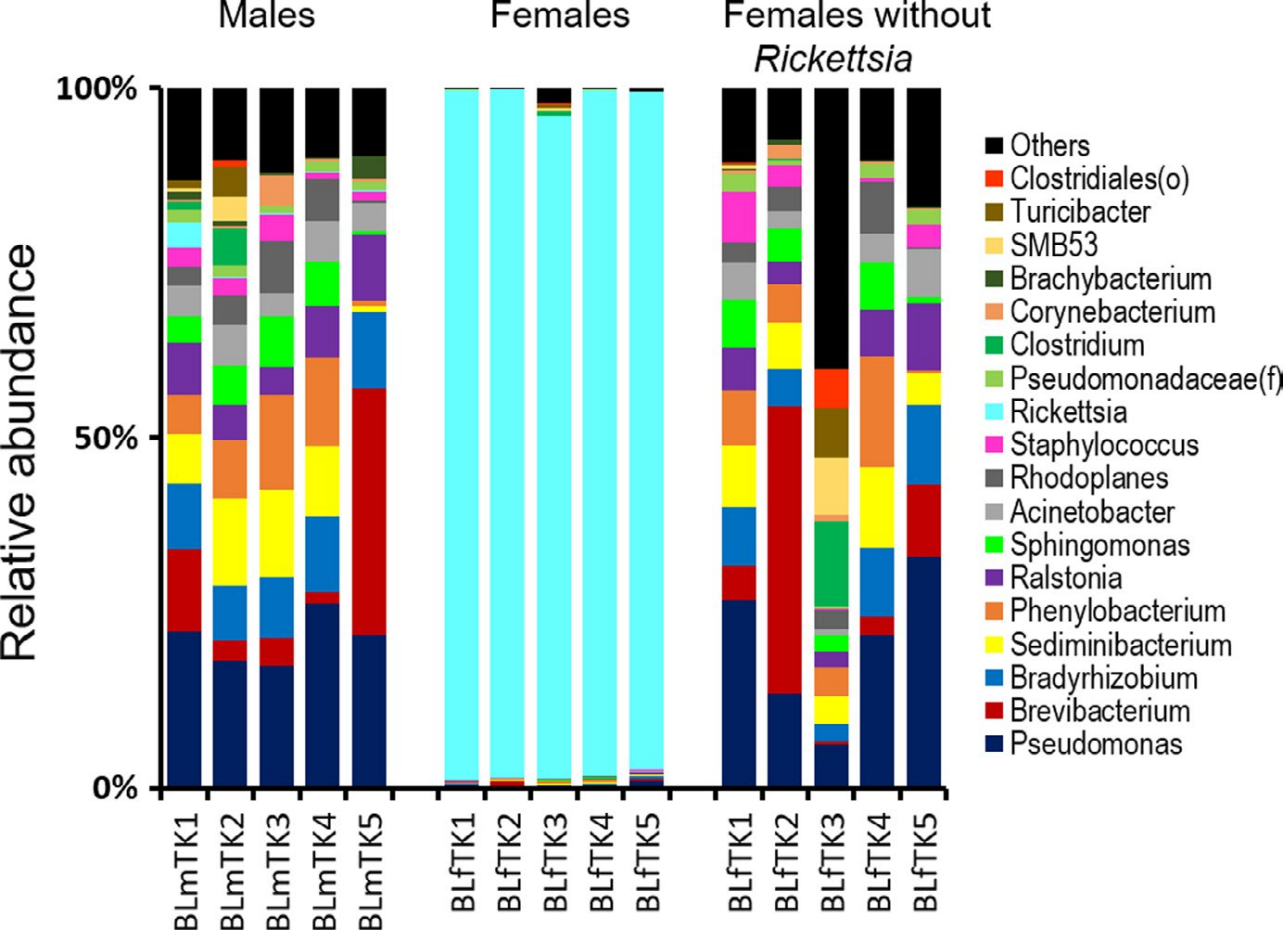
Bacterial microbiome in baseline populations of the colony‐reared *Ixodes scapularis* adult ticks. Each bar represents an individual whole tick sample (identified on *x*‐axis), where different colors indicate the relative proportions of various bacteria characterized to the genus level (wherever possible) based on 97% OTU similarity threshold of bacterial 16S V4 rRNA gene using Greengenes reference database. Baseline ticks were frozen upon receipt and not exposed to any temperature incubations. (Left panel, male ticks; middle panel, female ticks; right panel, female ticks after in silico removal of *Rickettsia*)

The microbiome results showed that the bacterial community composition differed by gender. Male ticks had a more complex bacterial microbiome than females. At the phylum level, Proteobacteria comprised the highest relative abundance across all baseline male ticks (range = 54.7%–83.4%, mean = 67.4%), followed by Actinobacteria (4.3%–41.4%, 16.6%), Bacteroidetes (1.6%–13.2%, 9.3%), and Firmicutes (1.7%–17.8%, 6.7%). By comparison, baseline females had the OTUs assigned almost entirely to a single phylum Proteobacteria (range = 95.9%–99.6%, mean = 98.6%) (Supporting Information Figure S4). Consistent with the pattern of phylum‐level results, 21 genera (plus one order‐level taxon, and two family‐level taxa) were found with a relative abundance of ≥1% in at least one of the baseline male samples, while the females had only two genera with 1% or greater abundance (Figure 2). The top 10 genera (mean relative abundances) in baseline male tick samples were comprised of *Pseudomonas* (21.2%), *Brevibacterium* (11.2%), *Bradyrhizobium* (9.5%), *Sediminibacterium* (8.6%), *Phenylobacterium* (8.2%), *Ralstonia* (6.6%), *Sphingomonas* (4.8%), *Acinetobacter* (4.6%), *Rhodoplanes* (4.1%), and *Staphylococcus* (2.3%). A small proportion of the male microbiome constituted genera, such as *Corynebacterium, Brachybacterium*,* Rickettsia,* and others. In contrast, the microbiome of baseline female ticks was dominated entirely by the genus *Rickettsia* (range = 94.6%–98.5%), which had a significantly (Kruskal–Wallis test *p *=* *0.009) lower relative abundance (0.1%–3.7%) in males.

Because the microbiomes of female ticks were completely dominated by amplicons derived from the rickettsial endosymbiont (i.e., *R. buchneri*) known to occur in this tick species, and because this endosymbiont resides primarily in the ovaries (Kurtti et al., 2015), we performed in silico removal of *Rickettsia* sequences from the female data to assess the underlying bacterial microbiome of the female ticks. Removal of *Rickettsia* from baseline female data sets resulted in an average of 4,155 reads per sample (range = 1,809–6,600). *Rickettsia‐*free baseline female data revealed a total of 34 taxa (including 25 genera) with 1% or greater abundance in at least one of the samples. The bacterial community composition in *Rickettsia‐*free baseline females (Figure 2 right panel) was consistent with those observed among full profiles (i.e., *Rickettsia* included) of baseline male ticks (Figure 2 left panel). Removal of *Rickettsia* sequences from the baseline male datasets did not show any difference in the overall community composition when compared to those with all sequences (Supporting Information Figure S5).

### 3.3 Bacterial microbiome of *I. scapularis* ticks incubated at different temperatures

The phylum Proteobacteria had the highest abundance in all male ticks incubated at 4°C for 10 days (mean = 71.1%), 20°C for 10 days (78.3%), 30°C for a week (81.9%), and 37°C for 5 days (88.2%) (Supporting Information Figure S4a), which was preceded by the phyla Actinobacteria, Bacteroidetes, and Firmicutes. On the other hand, Actinobacteria was highly abundant among male ticks held at 30°C for 8–10 days (mean = 61.9%) and 37°C for 7–9 days (98.9%). In contrast, Proteobacteria was the dominant phylum across all temperatures in the female samples, except a few individuals incubated at 30°C for 10 days and 37°C for 9–10 days (where ~50% of the total bacterial phyla was represented by Actinobacteria) (Supporting Information Figure S4b).

**Figure 3:**
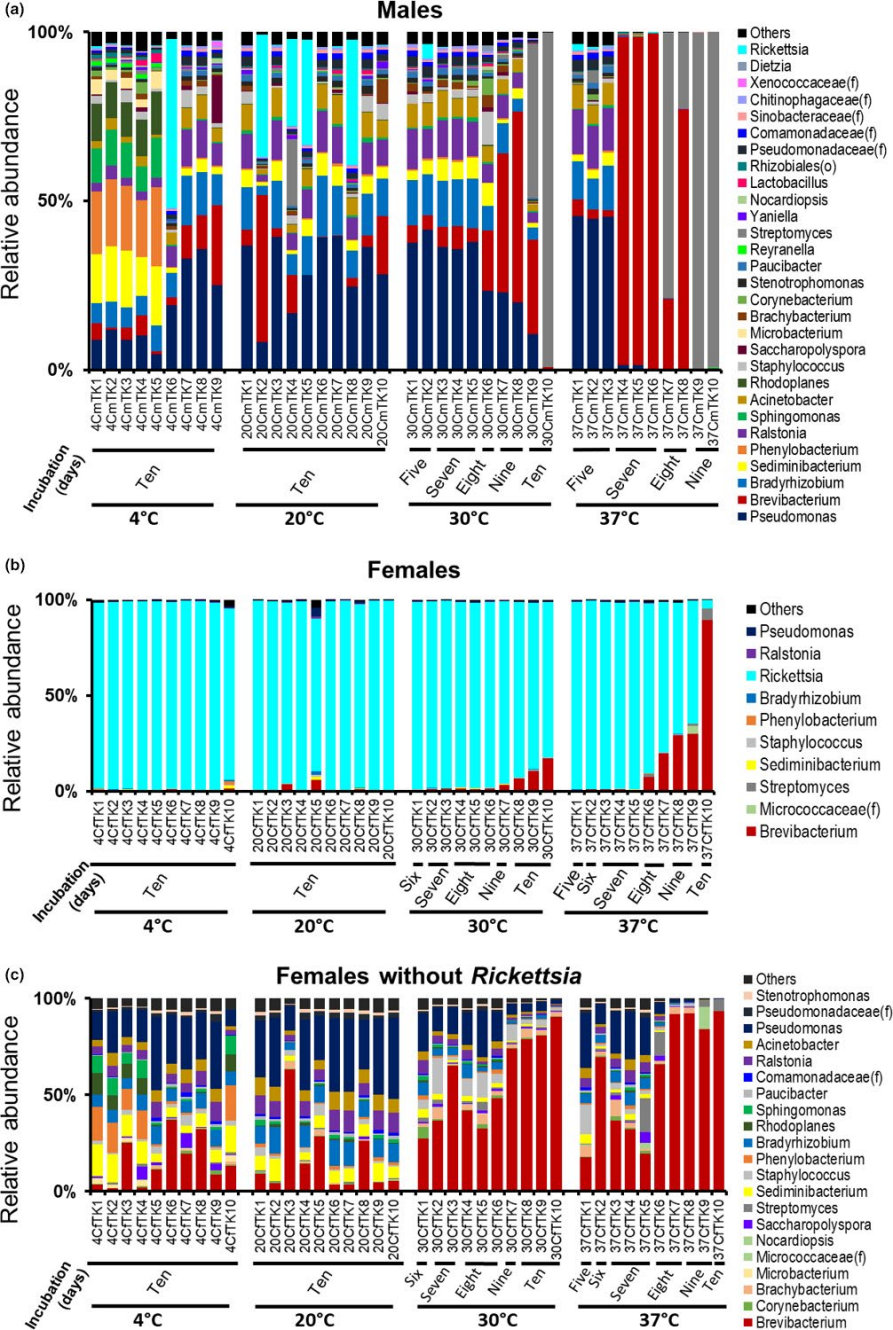
Bacterial microbiomes of colony‐reared *Ixodes scapularis* adult (a) male and (b) female ticks incubated at 4, 20, 30, and 37°C, for up to 10 days. (c) Bacterial abundance in female ticks shown in (b) after in silico removal of *Rickettsia*. Each stacked bar represents a tick sample (identified on *x*‐axis) whose DNA was extracted individually from the whole tick. Colors represent genus‐level (wherever possible) taxonomic composition of bacteria with size reflecting their relative abundances

In the male ticks, *Pseudomonas, Phenylobacterium, Sediminibacterium, Bradyrhizobium, Brevibacterium, Ralstonia, Rhodoplanes, Sphingomonas, Acinetobacter,* and *Staphylococcus* constituted the top 10 genera at 4°C, accounting for more than three‐fourths of the bacterial community (Figure 3a). In addition, many of these bacteria were also the major genera in the males held at 20°C, where forty percent of the tick population had high abundance of *Rickettsia* (mean = 32.6%). Furthermore, *Pseudomonas* had the highest relative abundance in males held at 30°C for 5–7 days (mean = 37.9%), whereas *Brevibacterium* (48.8%) and *Streptomyces* (72.3%) were the dominant genera in the day 9 and day 10 samples. Even at 37°C, *Pseudomonas* represented the most highly abundant genus (45.2%) in day 5 males and was found in all other male samples held at 37°C, but with a low relative abundance (<0.3%). *Brevibacterium* (97.9%) and *Streptomyces* (99.3%) were the two genera that entirely dominated the day 7 and 9 male samples, respectively (Figure 3a). Bacterial microbiomes of *Rickettsia*‐deleted male samples (Supporting Information Figure S6) did not differ in their composition when compared to those with their full profiles.

In female ticks with all sequences, *Rickettsia* was the only dominant genus (64.5%–99.5%) across all temperature treatments regardless of the duration of incubation, except one sample incubated at 37°C for 10 days which contained *Brevibacterium* in the highest proportion (89.2%) (Figure 3b). Female ticks incubated at 30°C for 10 days had an average relative abundance of *Brevibacterium* of 11.4% while those kept at 37°C for 8 and 9 days had 13.6% and 29.5% *Brevibacterium*, respectively (Figure 3b). Analyses of the underlying bacterial microbiome composition remaining after in silico deletion of *Rickettsia* sequences from the female data set revealed a diverse bacterial community in the female ticks with the dominance of *Brevibacterium* in both 30 and 37°C treatment groups (Figure 3c). These results were consistent with those seen among male samples.

### 3.4 Comparison of the bacterial microbiome in *I. scapularis* ticks incubated at different temperatures with that of baseline ticks

There was no significant change in the bacterial composition of *I. scapularis* ticks incubated at 4 and 20°C (at constant humidity of >80%) for 10 days, compared to that of the baseline samples. However, the microbiome composition of the baseline *I. scapularis* ticks was different in comparison with those incubated at 30°C for more than a week or at 37°C for more than 5 days. The differences were more prominent in male ticks compared to the full female profiles (i.e., *Rickettsia* included). The phylum Proteobacteria, which represented the highest relative abundances across all baseline samples, showed a significantly decreased (Kruskal–Wallis test *p *=* *0.004) mean abundance (from 67.4% to 1%) in male ticks incubated for 7–9 days at 37°C. On the contrary, males held at 37°C for 7–9 days showed a significant increase (*p *=* *0.004) in relative abundance of the phylum Actinobacteria (from a mean relative abundance of 16.6% to 98.9%) (Supporting Information Figure S4).

In male ticks held at 37°C, the relative abundance of *Pseudomonas* was significantly decreased (Kruskal–Wallis test *p *=* *0.017) from day 5 (mean = 42.5%) to day 9 (average for 7–9 days = 0.5%). A significant difference (*p *=* *0.004) was also observed when abundances of *Pseudomonas* in baseline males (21.2%) were compared to that of the males held at 37°C for more than 5 days (0.5%).

Relative percent of *Brevibacterium* dramatically increased (from the baseline average of 11.2% to 97.9%, Kruskal–Wallis test *p *=* *0.025) in male ticks incubated at 37°C for 7 days, but show a decreased abundance in day 8 samples (mean = 48.9%), and almost 0.3% abundance by day 9 (Figures 2 and 3a). In addition, *Streptomyces,* with a very low abundance at baseline (0.5%), was highly represented in day 8 samples (mean = 50.5%) and dominated the bacterial community in ticks held at 37°C for 9 days. The abundance of the genus *Streptomyces* was significantly different when the unexposed (baseline) male ticks were compared to the males incubated for 8–9 days at 37°C (*p *=* *0.014), but not in baseline males versus the males held at 37°C for 5 days (*p *=* *0.297).

The mean abundance of *Rickettsia,* the only dominant genus across all females, slightly decreased from 97.2% (baseline) to 92.1% in 8–10 days at 30°C. In addition, there was a relatively low percentage of *Rickettsia* (75%) in females incubated at 37°C for 8–9 days, which corresponds to an increase in abundance of *Brevibacterium* (from a baseline value of 11.2% to 21.6%). Based on PERMANOVA, the bacterial community of males incubated at 37°C showed a significant difference compared to baseline samples (Adonis statistic, *F* = 6.0044, *p *=* *0.001).

**Figure 4:**
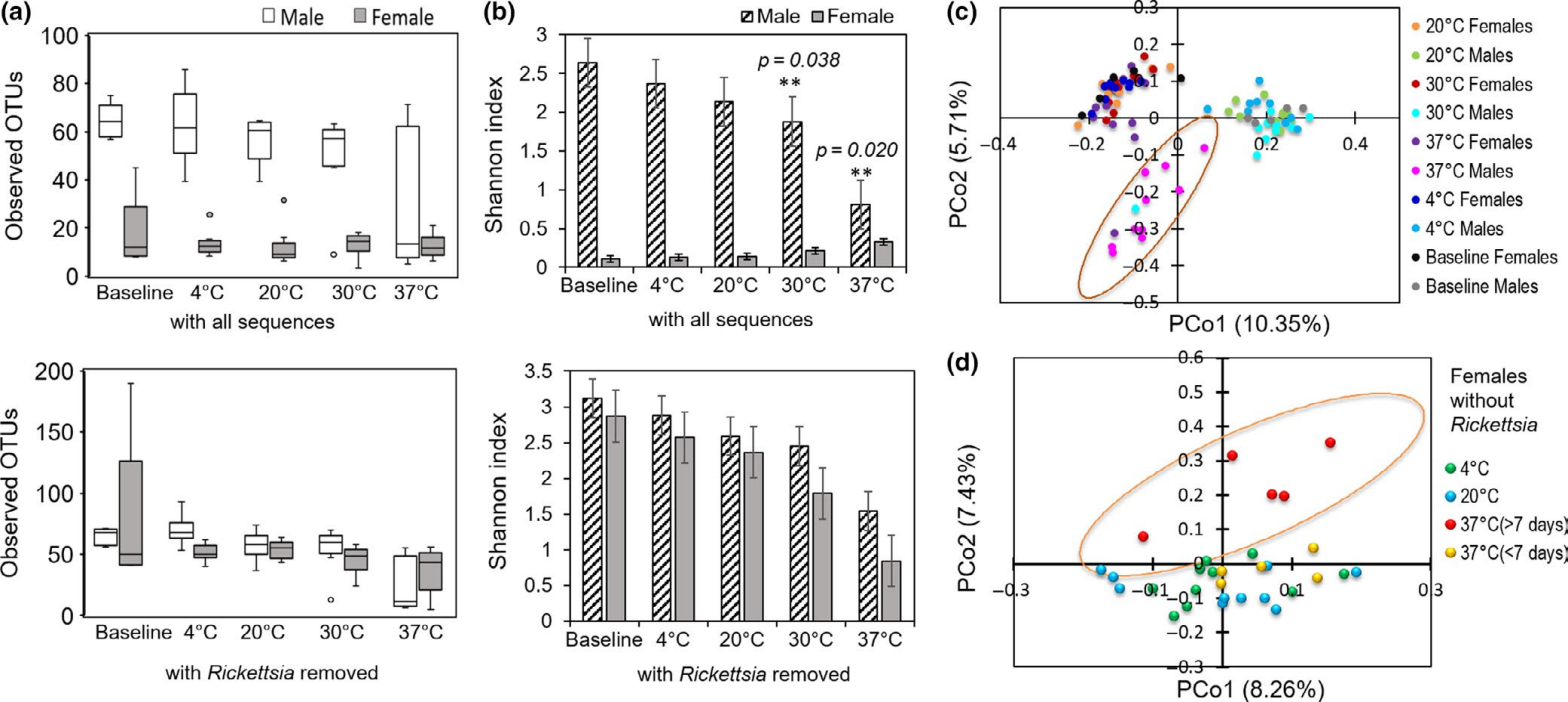
Effect of temperatures on diversity of *Ixodes scapularis* microbiomes as measured by (a) observed number of OTUs (upper panel, with all sequences; lower panel, after in silico removal of *Rickettsia*), (b) Shannon index (upper panel, with all sequences; lower panel, with *Rickettsia* removed). (c) PCoA plots of unweighted UniFrac distances of all the samples and (d) females without *Rickettsia* sequences. The samples inside the ellipses are clustered separately from the remaining groups (95% confidence intervals). Each dot in the PCoA plot represents the bacterial microbiome of an individual tick. **Benjamini–Hochberg‐corrected *p‐*values at 0.05 FDR

In *Rickettsia‐*free female data, dominance of *Brevibacterium* was prominent in both 30°C (mean = 57.6%) and 37°C (60.3%) treatment groups, compared to the baseline females (11.9%). Multiple pairwise comparisons using the Benjamini–Hochberg (BH) procedure at 0.05 FDR revealed that relative abundance of *Brevibacterium* in *Rickettsia*‐deleted baseline female samples was significantly different than those incubated for more than 7 days at both 30°C (*p *=* *0.02) and 37°C (*p = *0.001). In addition, a statistically significant difference (BH corrected *p *=* *0.02) was found when the relative abundance of *Brevibacterium* in *Rickettsia‐*free female ticks treated at 37°C for up to 7 days was compared to those incubated at 37°C for more than 7 days. This also resulted in separation of clusters of the *Rickettsia‐*deleted female ticks incubated for >7 days at 37°C from those incubated for 7 or fewer days in a PCoA plot (Figure 4d).

### 3.5 Effect of temperature on the bacterial diversity of ticks

For diversity analyses, the data with all sequences were normalized by rarefying to a depth of 2,399 reads. Our analyses showed that male ticks with their full bacterial profiles exhibited a higher bacterial richness (number of observed OTUs) across all conditions compared to the females (Figure 4a, upper panel). Similar results were obtained with other measures of alpha‐diversity (see Supporting Information Table S1). However, the diversity (Shannon index) was generally decreased in male ticks across all temperature groups, compared to the baseline (Figure 4b, upper panel). Furthermore, males incubated at 30 and 37°C exhibited significantly different Shannon index values compared to the baseline (Mann–Whitney test BH‐corrected *p* values 0.038 and 0.020, respectively). Moreover, the diversity in males decreased with increased length of incubation at 37°C, as evidenced by a significantly different (*p *<* *0.05) Shannon index in the male ticks incubated for more than 5 days at 37°C when compared to those treated at 37°C for 5 days and the baseline controls. The PCoA of unweighted UniFrac distances of bacterial communities showed that the first two axes (PCo1 and PCo2) explained 10.35% and 5.70% of the variation in the data, respectively, with the 37°C male samples clustering separately from others indicating that male ticks incubated at 37C° had a distinct bacterial composition compared to other ticks (Figure 4c).

For diversity analyses of *Rickettsia‐*deleted data sets, rarefaction was performed at a depth of 1,034 reads (which resulted in exclusion of three female samples, one in 4°C and two in 20°C groups, for further analysis). Bacterial richness (observed OTUs) and diversity (Shannon index) in *Rickettsia*‐free female ticks were similar to those of the males across all groups, as shown in the lower panels of Figure 4a,b, respectively. When the *Rickettsia‐*free dataset was rarefied to 2,399 reads, similar results were obtained despite the reduction in sample size (data not shown). The PCoA of the unweighted UniFrac distances of the female bacterial communities after in silico removal of *Rickettsia* revealed a significantly different bacterial diversity at 37°C (>7 days) compared to that of the 20°C treatment group (AMOVA test *p *<* *0.005), which clustered separately in the plot (Figure 4d). Likewise, the weighted UniFrac distances of the *Rickettsia*‐free female ticks incubated at 37°C had a significantly different bacterial diversity compared with that of the 4°C (AMOVA test *p = *0.00010) and 20°C (*p *=* *0.0001) treatment groups.

## 4 DISCUSSION

Ticks are important vectors of many human and animal pathogens. In addition, they carry numerous endosymbionts and commensals, which may provide nutrient supplements to the tick and also affect vector competence (reviewed in (Narasimhan & Fikrig, 2015; Bonnet et al., 2017; Clay & Fuqua, 2010)). Studies have reported that the tick microbial community is variable depending on several factors such as source of host blood meal (Rynkiewicz, Hemmerich, Rusch, Fuqua, & Clay, 2015), feeding status (Menchaca et al., 2013; Swei & Kwan, 2017; Zhang et al., 2014), tick species, life stage, gender, and geographical origin (Carpi et al., 2011; Van Treuren et al., 2015; Williams‐Newkirk, Rowe, Mixson‐Hayden, & Dasch, 2014). However, very few studies have focused on the tick microbiome differences based on environmental conditions such as seasons of collection (Lalzar, Harrus, Mumcuoglu, & Gottlieb, 2012) or the immediate environment (Menchaca et al., 2013).

In this study, we investigated the effect of environmental temperature on the bacterial microbiome of *I. scapularis* ticks, the primary Lyme disease vector in North America. The findings of the present study help enhance our understanding of the impacts of changes in environmental temperature in the shaping of microbial community structure in tick vectors, which in turn may be important for predicting the potential effect of warming climate on pathogen acquisition and vector competence in arthropod vectors and understanding factors influencing the transmission dynamics of vectorborne diseases.

Despite the same age (~4 months), rearing environment, and blood meals (in early life stages), the adult male and female *I. scapularis* ticks exhibited a distinct bacterial community structure. Male ticks displayed more heterogeneous bacterial communities than females, which contained almost exclusively *Rickettsia* (Figure 2). The high relative abundance of *Rickettsia* in the female ticks has been reported previously (Kurtti et al., 2015). Other than *Rickettsia*,* Pseudomonas* was the only genus found with a relative abundance of ≥1% in females, even though 37 genus‐level taxa (plus more higher level taxonomic groups) were present. In contrast, baseline male ticks had a very low abundance of *Rickettsia* (mean = 0.9%). Besides *Rickettsia,* 21 genera (plus one order‐level taxon, and two family‐level taxa), were found with ≥1% relative abundance in at least one of the baseline male samples. Relatives of many of these bacteria identified in male ticks (*Pseudomonas*,* Brevibacterium*,* Bradyrhizobium*,* Sediminibacterium*,* Phenylobacterium Ralstonia*,* Sphingomonas*,* Acinetobacter*,* Rhodoplanes*) are associated with soil, water, and plants, suggesting that these bacteria may be acquired from their environments and maintained through molting.

Although microbial community diversity is different between laboratory‐reared and wild‐caught ticks (Heise, Elshahed, & Little, 2010), Zolnik et al. (2016) also found a high relative abundance of *Rickettsia* in field‐collected whole adult female blacklegged ticks (mean = 97.9%), but comparatively low abundance in the males. Furthermore, *Rickettsia* was abundantly found in a laboratory‐reared larval population of *I. scapularis* (Narasimhan et al., 2014; Zolnik et al., 2016). Another study on *I. scapularis* also reported a low abundance of *Rickettsia* in males, possibly due to loss of the *Rickettsia* endosymbionts while transitioning through life stages in males (Noda, Munderloh, & Kurtti, 1997). These reports were corroborated by the presence of *Rickettsia* in ovaries of female ticks, and its absence in the testes of adult males (Noda et al., 1997). Thus, our findings of *Rickettsia* in both male and female blacklegged ticks, with high levels in the females, are in concordance with previous studies (Van Treuren et al., 2015; Zolnik et al., 2016).

*Rickettsia*, which contains a number of tickborne pathogenic species, including *Rickettsia rickettsii, R. japonica, R. akari* (Azad & Beard, 1998), and *R. parkeri* (Paddock et al., 2004)*,* also contains many non‐pathogens. The rickettsial endosymbiont of *I. scapularis* and *R. buchneri* (Kurtti et al., 2015) has been reported to provide a source of vitamins to the tick (Hunter et al., 2015). In addition, the *Rickettsia* endosymbiont of *I. pacificus*, closely related to *I. scapularis,* has been shown to contribute to the synthesis of folic acid (Hunter et al., 2015). Because the microbiomes of female ticks were entirely dominated by the *R. buchneri*, the endosymbiont known to occur in *I. scapularis*, and because this endosymbiont resides primarily in the ovaries, we removed *Rickettsia* sequences from the female data sets to further explore the underlying gut microbiome of the female ticks. There are several approaches to achieve the goal of minimizing or removing the effects of endosymbiotic bacteria: deeper sequencing, bioinformatic (in silico) removal of the sequences from the dataset, or using primers for blocking the amplification of unwanted target (Gofton et al., 2015; Greay et al., 2018). In silico removal of *Rickettsia* from our female dataset revealed a total of 34 taxa (including 25 genera) with 1% or greater abundance in at least one of the samples, consistent in complexity with those seen among the males, suggesting that the high numbers of *Rickettsia* were masking the underlying diversity in female ticks. Our findings of a more complex bacterial community composition in female *I. scapularis* after *Rickettsia* are removed indicates that this approach may be useful to understand the underlying gut microbiome in the whole female tick samples.

In our experimental study, all ticks survived 10 days at 20°C. One tick each was found dead on the 10th day for both male and female groups incubated at 4°C. By contrast, ticks of both sexes began dying at day 5 in both the 30 and 37°C, with slightly better survivorship among females. This is likely due to heat stress, with increased female body size possibly providing some level of protection. A recent study by Ginsberg et al. (2017) also showed a decreased survival of immature larval *I. scapularis* at 32.2°C compared to 22.2°C with a relatively similar humidity of ~85%.

We found that colony‐reared *I. scapularis* adult ticks incubated for 10 days at 4 and 20°C (with >80% humidity) showed no significant change in bacterial community composition when compared to the untreated baseline microbiomes, suggesting that these temperatures may have very little to no effect in the adult tick microbiomes. We also found a fairly‐standard bacterial community in the first days of incubation at 30 and 37°C, but the community complexity of the ticks drops dramatically upon prolonged incubation, particularly after more than a week at 30 and longer than 5 days at 37°C. Our finding of decreased relative abundance of *Pseudomonas* in male ticks held at 30°C for more than a week and at 37°C for more than 5 days, with a concomitant increase in relative proportion of *Brevibacterium*, suggested the potential impact of warmer temperatures in eliminating certain groups of bacteria while favoring others. This was also seen with the genus *Streptomyces*, which showed a significant increase in mean relative abundance in male ticks incubated across high temperatures (from the 0.5% at baseline to 72.3% at 30°C for 10 days, to 99.3% at 37°C for 9 days). Our findings of significantly higher abundance of *Brevibacterium* in *Rickettsia‐*free female data treated at 37°C for more than 7 days, compared to those incubated for up to 7 days and the *Rickettsia‐*deleted baseline female ticks, further support the idea that warmer temperature incubation may select for certain groups of bacteria over others, with unknown effects on vector competence for disease‐causing organisms. Taken together, these data provide evidence that the bacterial microbiome of *I. scapularis* changes upon extended incubation at 30 and 37°C.

In addition, the microbiome changes in *I. scapularis* ticks could not be directly attributed to the death of the ticks, because even at the same temperature treatment (e.g., 37°C male groups), the microbiome of the ticks that died early (less than 6 days) had a different bacterial composition than those that died later (by day 8 or 9), and secondly, the microbiome of the ticks that died early (e.g., less than 7 days at 30°C) had a similar microbiome to that of the baseline ticks (which were live ticks that were preserved at −20°C immediately upon receipt in our laboratory).

In both cases, with all sequences considered and or with *Rickettsia* removed datasets, we found a general decrease in the bacterial richness and diversity of the male ticks as the temperature increased from 4 to 37°C. With full bacterial profiles considered, males incubated at 37°C for more than 5 days showed a significant decrease (*p *<* *0.05) in the bacterial diversity compared to those treated at 37°C until 5 days and the baseline population, most likely due to the higher abundances of either *Brevibacterium* or *Streptomyces* (see Figure 3a). Because the nature of the experimental design provided information on relative abundances rather than absolute numbers, it is not known whether the shift in population resulted from an increase in absolute numbers of *Brevibacterium* and/or *Streptomyces* decreases in absolute numbers of other bacteria or some combination of the two. *Streptomyces* spp. are notable for their natural production of a number of antibiotics. It is interesting to speculate whether this may also have played a role in the microbial population shifts among these ticks.

The low number of observed OTUs in baseline females (with all sequences considered), as well as across all temperature treatments (see Figure 4a, upper panel), is consistent with a dominance of *Rickettsia* throughout all groups. Our results are based on the analysis of the total microbiome of the whole tick, so one could predict different results if various organs of the ticks were assessed separately. However, a previous study on field‐collected *I. scapularis* found the highest observed OTUs in whole females compared to the salivary glands and midguts (Zolnik et al., 2016), suggesting that our approach was relevant to assess the natural microbiome of the female ticks.

Later in silico removal of the sequences of *Rickettsia* from the female data revealed a shift in the remaining bacterial population and dramatic increases in relative *Brevibacterium* levels at 30 and 37°C (see Figure 3c), with significantly different microbiomes at 37°C compared to the 4 and 20°C treatments (Figure 4d), suggesting that the rickettsial abundance had been masking the effects of temperature on the underlying diversity of the remaining bacterial population in female *I. scapularis* ticks. This finding is also supported by the observation of a shift in the observed number of OTUs and the Shannon index in *Rickettsia*‐deleted female data (Figure 4a,b, lower panels) when compared to those females with all sequences (Figure 4a,b, upper panels). The bacterial community revealed after removal of the rickettsial sequences indicates that the microbiome of the female tick is similarly as diverse as males.

## 5 CONCLUSION

The bacterial microbiomes of colony‐reared *I. scapularis* were distinct between male and female ticks. The genus *Rickettsia* almost exclusively dominated the female ticks, whereas several environmental‐related genera were found in the males. In silico removal of *Rickettsia* sequences revealed a hidden bacterial community within the females, which was consistent in complexity with that of males. We found that the bacterial microbiome of *I. scapularis* males changes upon static incubation in controlled laboratory settings of 30°C for more than a week and 37°C for more than 5 days at a constant humidity of >80%. In addition, male ticks incubated at 30 and 37°C exhibited significantly different bacterial diversity (Shannon index) compared to the baseline population, and the change in diversity was dependent upon duration of exposure. *Rickettsia*‐free data also revealed a significantly different bacterial diversity in female ticks incubated at 37°C when compared to that of 4 and 20°C treatments. In summary, the environmental temperature can impact the tick bacterial microbiome of *I. scapularis* in a laboratory setting. Future studies on how environmental variables influence vector microbiome composition, and the possible ramifications of this on the ticks’ ability to carry and transmit pathogens, are needed to better understand the impact of climate change on risk and spread of tickborne and other zoonotic diseases.

## CONFLICT OF INTERESTS

The authors declare that they have no competing interests.

## AUTHORS’ CONTRIBUTIONS

ST and MSA conceived and designed the study. MSA supervised the study. ST prepared samples for sequencing, performed PCR and sequencing experiments YZ processed the data using mothur. ST, YZ, and MSA analyzed data. ST and YZ performed statistical analyses. ST drafted the initial manuscript, and all authors provided feedback and insights into the manuscript. All authors read, edited, and approved the final version of the manuscript.

## Data Availability

Raw sequence data can be accessed at the sequence read archive (SRA) of the National Center for Biotechnology Information (NCBI) under the project PRJNA471905.
