# Supplementary material for: Effects of temperature on bacterial microbiome composition in Ixodes scapularis ticks
Source: Microbiologyopen. 2018 Sep 21;8(5):e00719. doi: 10.1002/mbo3.719 (PMC6528569; doi:10.1002/mbo3.719)
Supplement: Supplementary file 1 [file MBO3-8-e00719-s001.pdf]

APPENDIX: Supplemental materials

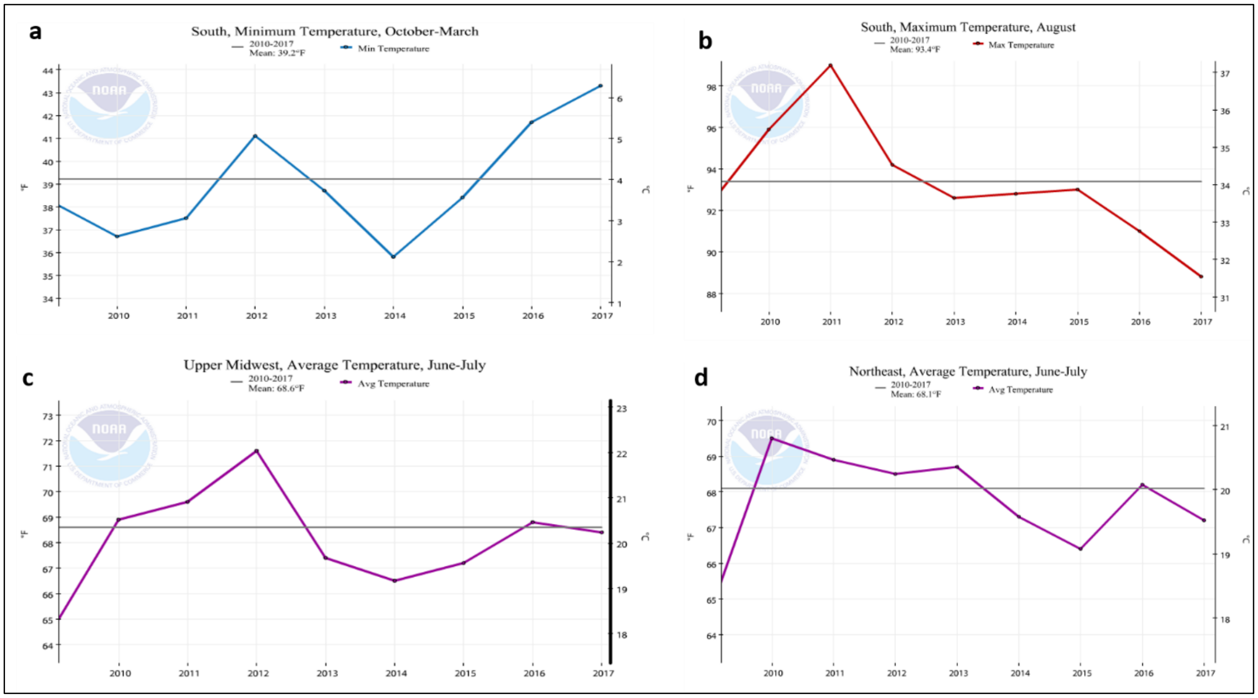

Figure S1

Temperatures in Upper Midwest, Northeast and South US Climatic Regions during 2010-2017.

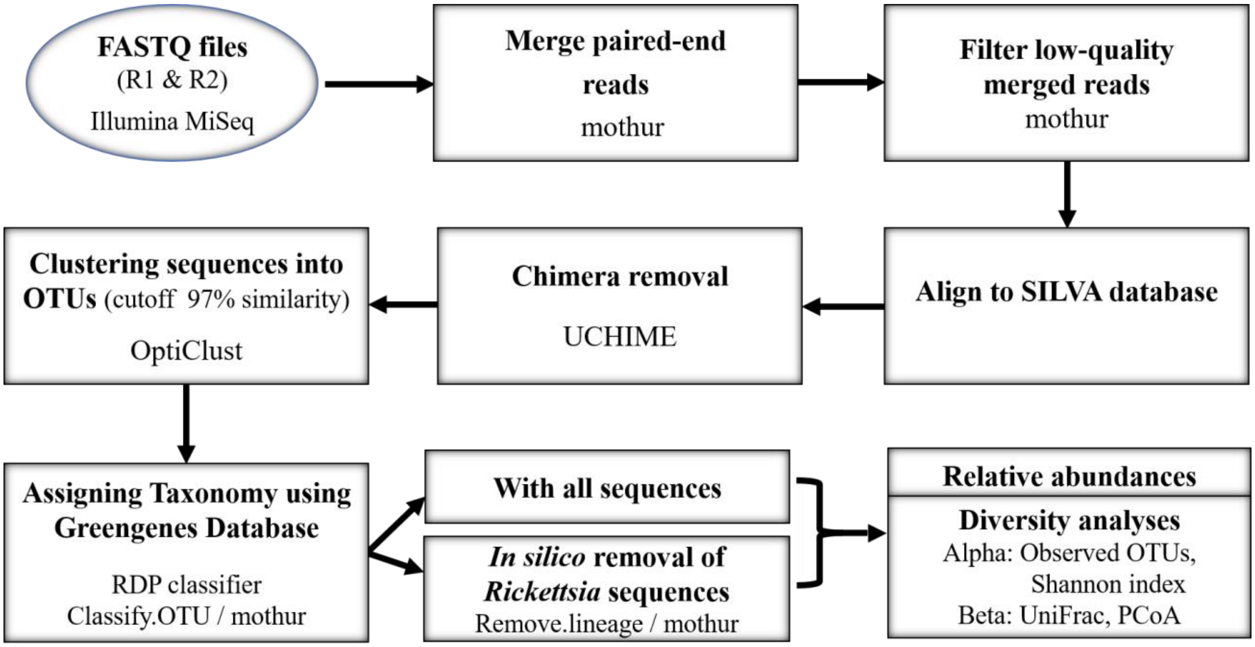

Figure S2

Bioinformatics workflow for processing of 16S rRNA gene sequences.

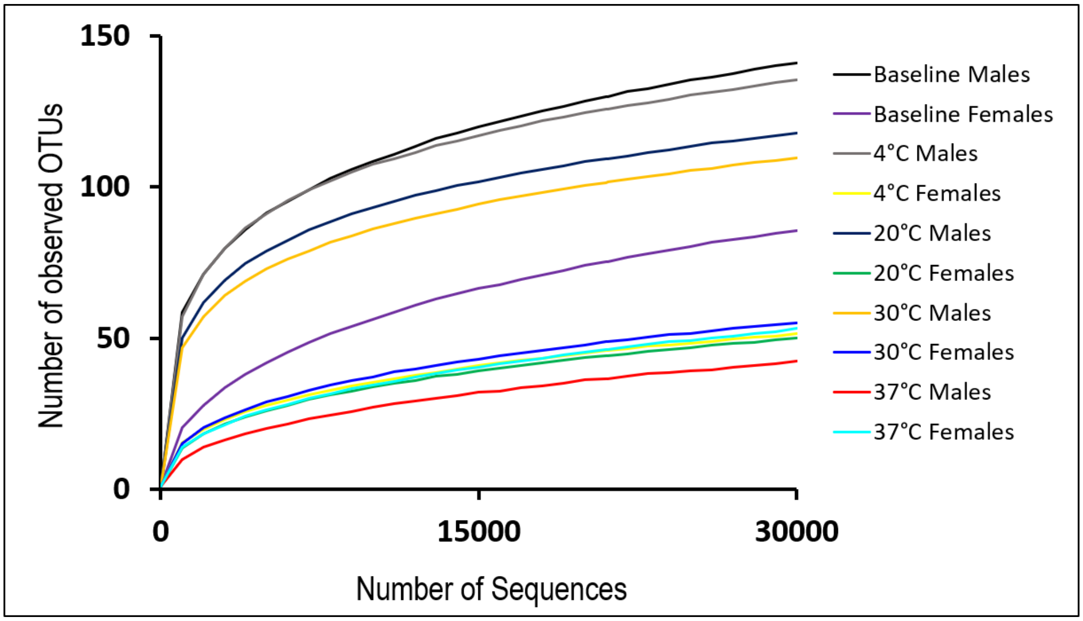

Figure S3

Rarefaction curves of observed OTUs versus increasing number of sequence**.** The curves represent the average of 30 iterations sampled every 1,000 sequences starting from 1,000 to 30,000 sequences.

Table S1

Good’s Coverage and other measures of alpha diversity per gender at baseline and various temperatures. (ACE= abundance-based coverage estimator; BL= baseline ticks; 4C, 20C, 30C and 37C denotes the ticks incubated at 4ºC, 20ºC, 30ºC and 37ºC temperatures respectively; m= male; f= female)

Sample ID	Good’s Coverage (%)	ACE value	Inverse Simpson index
I. scapularis males			
BLmTK1	99.05	161.74	11.83
BLmTK2	99.34	110.76	14.96
BLmTK3	99.37	108.41	11.18
BLmTK4	99.28	108.94	7.25
BLmTK5	98.96	179.29	4.53
4CmTK1	98.88	167.19	12.10
4CmTK2	99.12	136.10	8.54
4CmTK3	99.16	126.22	12.13
4CmTK4	98.85	160.35	13.00
4CmTK5	99.13	130.61	7.36
4CmTK6	99.28	134.78	2.52
4CmTK7	99.31	112.04	6.22
4CmTK8	99.15	123.94	6.10
4CmTK9	99.39	104.11	3.45
20CmTK1	99.16	124.06	5.96
20CmTK2	99.37	112.54	3.14
20CmTK3	99.12	137.49	5.41
20CmTK4	99.37	100.84	6.83
20CmTK5	99.44	87.40	4.36
20CmTK6	99.23	111.54	5.13
20CmTK7	99.21	117.62	4.63
20CmTK8	99.29	114.04	4.60
20CmTK9	99.18	125.35	5.52
20CmTK10	99.28	108.42	6.90
30CmTK1	99.20	120.70	4.93
30CmTK2	99.24	116.03	4.80
30CmTK3	99.33	102.66	6.17
30CmTK4	99.37	97.58	4.15
30CmTK5	99.31	107.27	5.27
30CmTK6	99.32	111.55	8.78
30CmTK7	99.19	144.48	4.37
30CmTK8	99.32	101.00	2.38

Sample ID	Good's Coverage (%)	ACE value	Inverse Simpson index
30CmTK9	99.29	113.65	3.37
30CmTK10	99.79	94.79	1.03
37CmTK1	99.29	99.48	4.37
37CmTK2	98.86	199.13	4.63
37CmTK3	99.14	127.75	4.34
37CmTK4	99.63	89.99	1.08
37CmTK5	99.65	101.63	1.08
37CmTK6	99.90	49.05	1.01
37CmTK7	99.84	68.79	1.57
37CmTK8	99.76	108.37	1.59
37CmTK9	99.85	57.66	1.02
37CmTK10	99.86	45.18	1.02
I. scapularis females			
BLfTK1	99.73	71.12	1.03
BLfTK2	99.76	64.77	1.04
BLfTK3	98.78	262.57	1.09
BLfTK4	99.80	61.03	1.02
BLfTK5	99.82	69.76	1.02
4CfTK1	99.76	44.29	1.04
4CfTK2	99.75	46.08	1.05
4CfTK3	99.78	55.19	1.03
4CfTK4	99.80	70.48	1.02
4CfTK5	99.79	86.38	1.02
4CfTK6	99.72	88.45	1.05
4CfTK7	99.76	76.32	1.02
4CfTK8	99.77	70.66	1.03
4CfTK9	99.71	64.70	1.04
4CfTK10	99.67	68.38	1.21
20CfTK1	99.80	81.61	1.02
20CfTK2	1.00	74.64	1.02
20CfTK3	1.00	57.44	1.08
20CfTK4	1.00	69.46	1.03
20CfTK5	0.99	104.54	1.36
20CfTK6	1.00	73.63	1.02
20CfTK7	1.00	57.97	1.01
20CfTK8	1.00	62.46	1.07
20CfTK9	1.00	68.12	1.01
20CfTK10	1.00	88.11	1.02
30CfTK1	99.70	78.20	1.04
30CfTK2	99.92	11.46	1.01
30CfTK3	99.87	55.49	1.02
30CfTK4	99.69	75.31	1.08

Sample ID	Good's Coverage (%)	ACE value	Inverse Simpson index
30CfTK5	99.64	95.79	1.08
30CfTK6	99.65	88.23	1.07
30CfTK7	99.75	83.68	1.09
30CfTK8	99.69	84.36	1.14
30CfTK9	99.69	72.21	1.23
30CfTK10	99.74	64.03	1.36
37CfTK1	99.83	52.05	1.02
37CfTK2	99.80	92.15	1.04
37CfTK3	99.68	85.20	1.06
37CfTK4	99.73	94.22	1.04
37CfTK5	99.68	72.95	1.05
37CfTK6	99.64	69.71	1.28
37CfTK7	99.79	77.33	1.39
37CfTK8	99.71	75.06	1.72
37CfTK9	99.78	129.25	1.62
37CfTK10	99.88	22.16	1.25

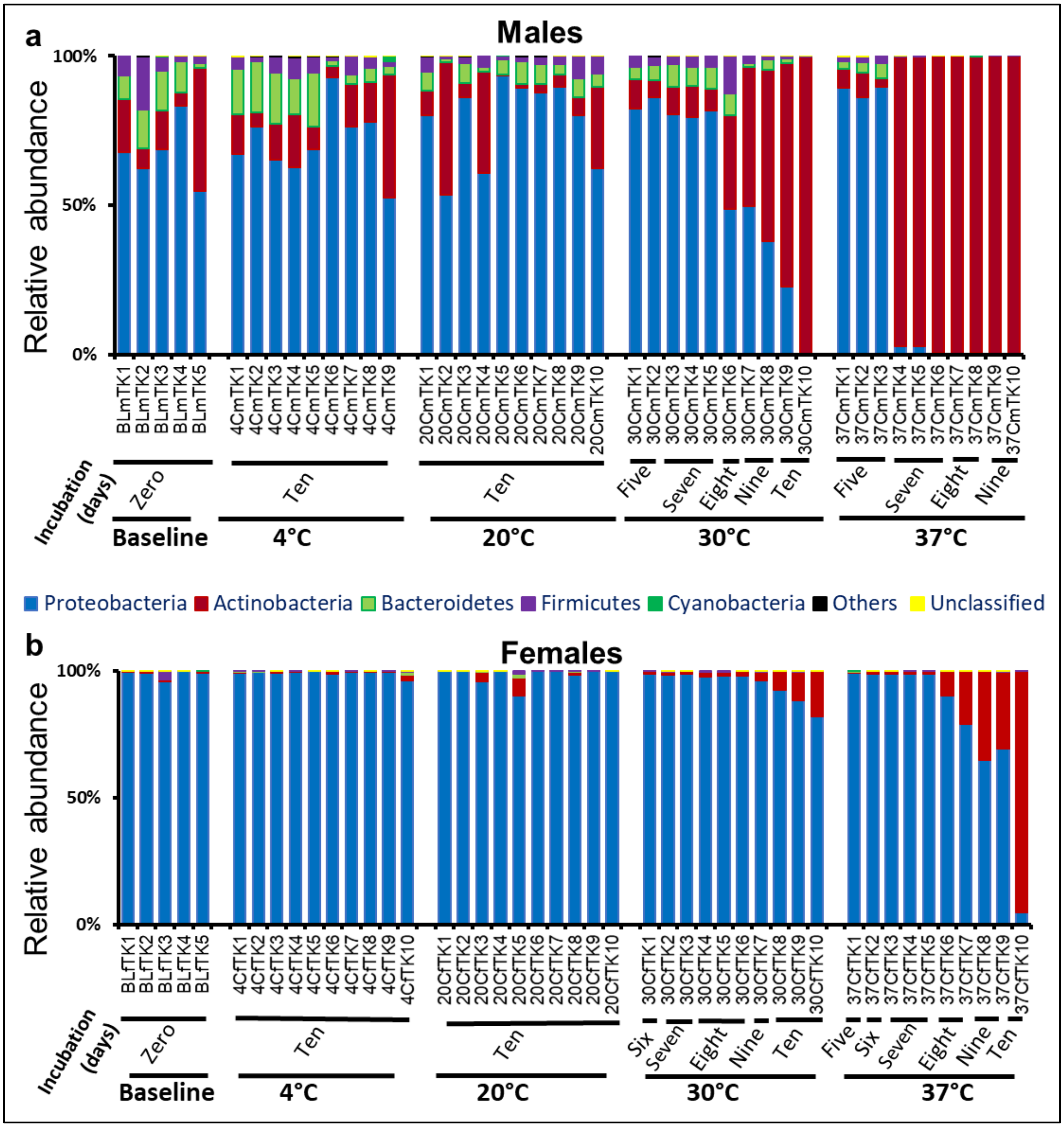

Figure S4

Phylum level bacterial microbiomes of *I. scapularis* male (a) and female (b) ticks. The microbiomes of both baseline and temperature-treated ticks (identified on x-axis) are shown in the stacked bar charts where each bar represents an individual tick.

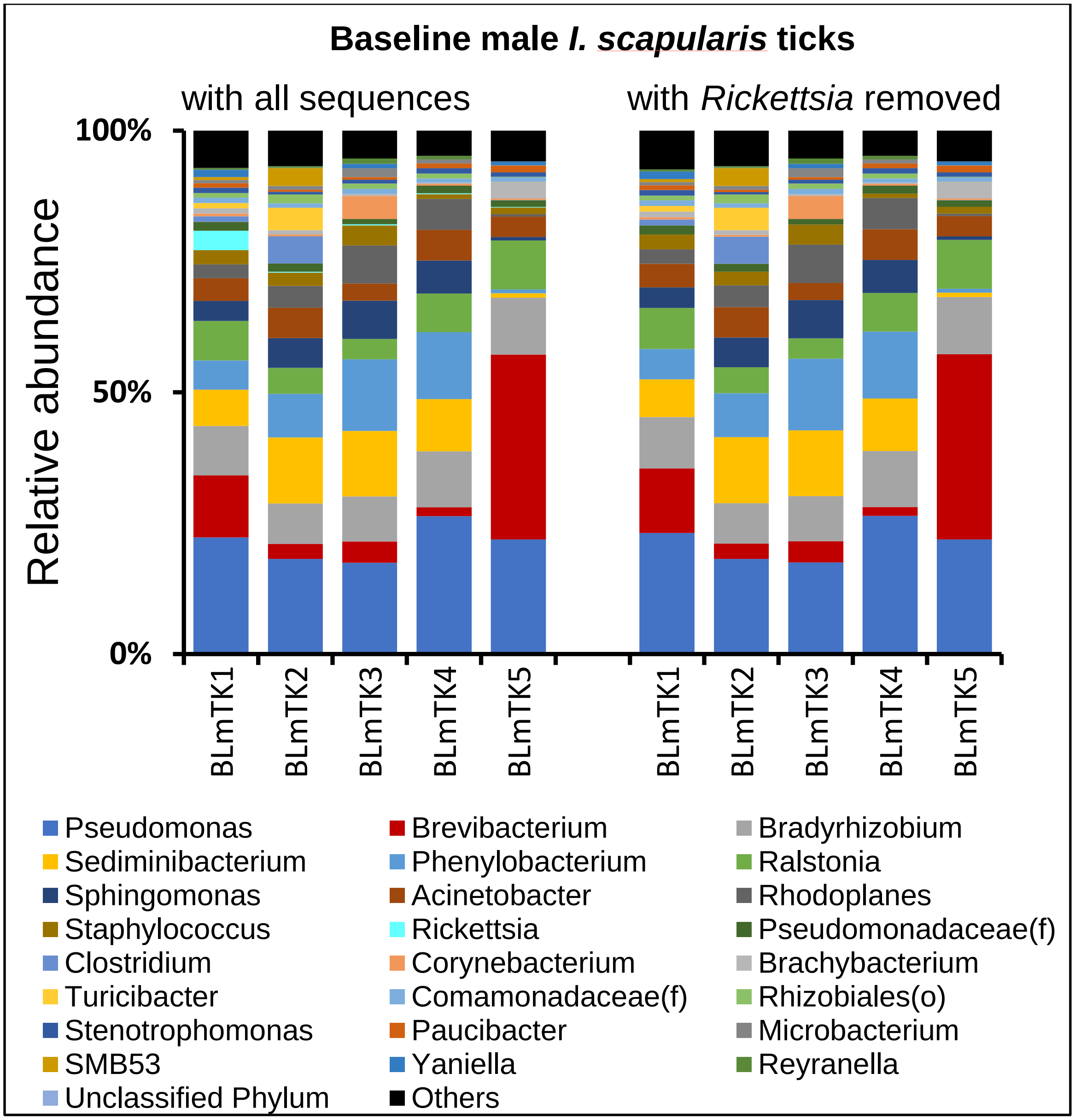

Figure S5

Relative abundance of bacterial taxa in colony-reared baseline *I. scapularis* males with all sequences (left panel) and with *Rickettsia* removed (right panel). Sequences with ≥ 1% relative abundance in at least one of the samples are reported individually, while remaining taxa are grouped together in an 'others' category.

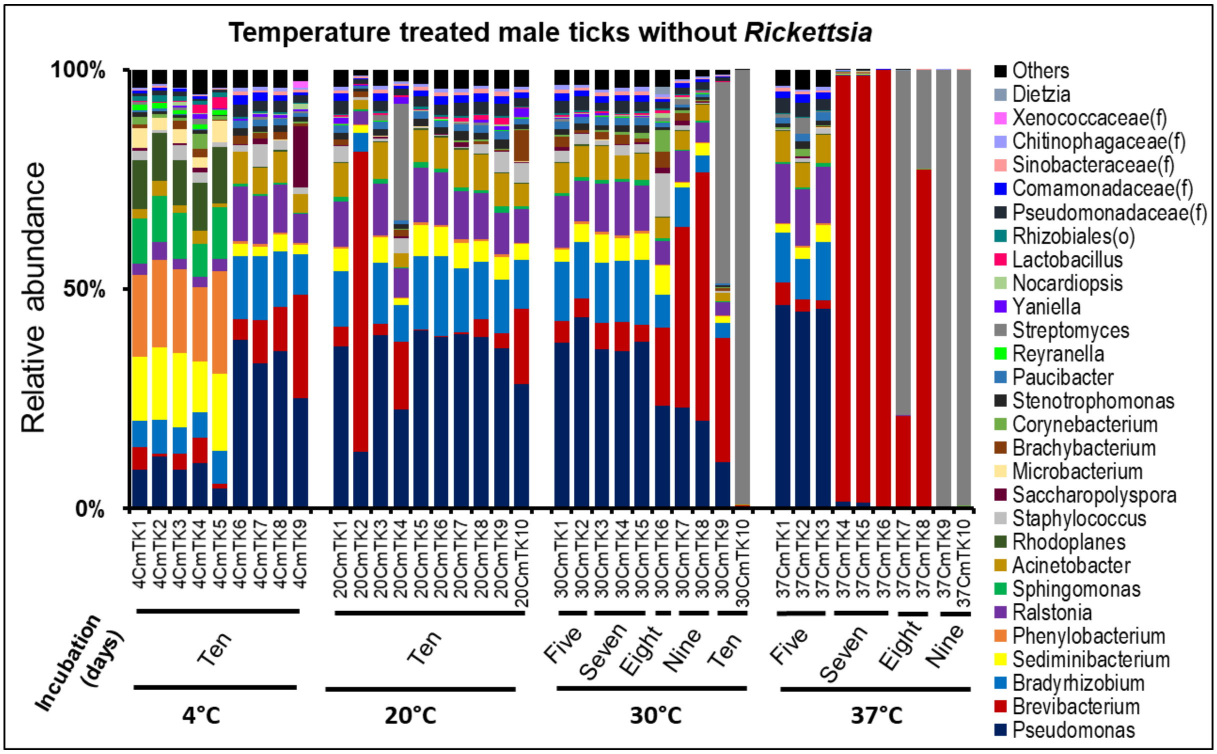

Figure S6

Bacterial microbiomes in male ticks shown in Fig. 3a after *in silico* removal of *Rickettsia*.

Each bar represents an individual tick sample.
